# Deep Sequencing of the *Trypanosoma cruzi* GP63 Surface Proteases Reveals Diversity and Diversifying Selection among Chronic and Congenital Chagas Disease Patients

**DOI:** 10.1371/journal.pntd.0003458

**Published:** 2015-04-07

**Authors:** Martin S. Llewellyn, Louisa A. Messenger, Alejandro O. Luquetti, Lineth Garcia, Faustino Torrico, Suelene B. N. Tavares, Bachar Cheaib, Nicolas Derome, Marc Delepine, Céline Baulard, Jean-Francois Deleuze, Sascha Sauer, Michael A. Miles

**Affiliations:** 1 The London School of Hygiene and Tropical Medicine, London, United Kingdom; 2 Molecular Ecology and Fisheries Genetics Laboratory, School of Biological Sciences, University of Wales, Bangor, Bangor, Gwynedd, United Kingdom; 3 Laboratório de Pesquisa da doença de Chagas, Hospital das Clínicas da Universidade Federal de Goiás, Brazil; 4 Facultad de Medicine, Universidad Mayor de San Simon, Cochabamba, Bolivia; 5 Institut de Biologie Integrative et de Systemes, Universite de Laval, Quebec, Canada; 6 Centre National de Génotypage, CEA, Evry, Paris, France; 7 Max Planck Institute for Molecular Genetics, Berlin, Germany; McGill University, CANADA

## Abstract

**Background:**

Chagas disease results from infection with the diploid protozoan parasite *Trypanosoma cruzi*. *T*. *cruzi* is highly genetically diverse, and multiclonal infections in individual hosts are common, but little studied. In this study, we explore *T*. *cruzi* infection multiclonality in the context of age, sex and clinical profile among a cohort of chronic patients, as well as paired congenital cases from Cochabamba, Bolivia and Goias, Brazil using amplicon deep sequencing technology.

**Methodology/ Principal Findings:**

A 450bp fragment of the trypomastigote TcGP63I surface protease gene was amplified and sequenced across 70 chronic and 22 congenital cases on the Illumina MiSeq platform. In addition, a second, mitochondrial target—ND5—was sequenced across the same cohort of cases. Several million reads were generated, and sequencing read depths were normalized within patient cohorts (Goias chronic, n = 43, Goias congenital n = 2, Bolivia chronic, n = 27; Bolivia congenital, n = 20), Among chronic cases, analyses of variance indicated no clear correlation between intra-host sequence diversity and age, sex or symptoms, while principal coordinate analyses showed no clustering by symptoms between patients. Between congenital pairs, we found evidence for the transmission of multiple sequence types from mother to infant, as well as widespread instances of novel genotypes in infants. Finally, non-synonymous to synonymous (dn:ds) nucleotide substitution ratios among sequences of TcGP63Ia and TcGP63Ib subfamilies within each cohort provided powerful evidence of strong diversifying selection at this locus.

**Conclusions/Significance:**

Our results shed light on the diversity of parasite DTUs within each patient, as well as the extent to which parasite strains pass between mother and foetus in congenital cases. Although we were unable to find any evidence that parasite diversity accumulates with age in our study cohorts, putative diversifying selection within members of the TcGP63I gene family suggests a link between genetic diversity within this gene family and survival in the mammalian host.

## Author Summary

*Trypanosoma cruzi*, the causal agent of Chagas disease in Latin America, infects several million people in some of the most economically deprived regions of Latin America. *T*. *cruzi* infection is lifelong and has a variable prognosis: some patients never exhibit symptoms while others experience debilitating and fatal complications. Available data suggest that parasite genetic diversity within and among disease foci can be exceedingly high. However, little is know about the frequency of multiple genotype infections in humans, as well as their distribution among different age classes and possible impact on disease outcome. In this study we develop a next generation amplicon deep sequencing approach to profile parasite diversity within chronic Chagas Disease patients from Bolivia and Brazil. We were also able to compare parasite genetic diversity present in eleven congenitally infants with parasite genetic diversity present in their mothers. We did not detect any specific association between the number and diversity of parasite genotypes in each patient with their age, sex or disease status. We were, however, able to detect the transmission of multiple parasite genotypes between mother and foetus. Furthermore, we also detected powerful evidence for natural selection at the antigenic locus we targeted, suggesting a possible interaction with the host immune system.

## Introduction

*Trypanosoma cruzi* is a kinetoplastid parasite and the causative agent of Chagas disease (CD) in Latin America. *T*. *cruzi* infects approximately 8 million people throughout its distribution and causes some 13,000 deaths annually [1]. Chagas disease follows a complex course. Infection, often acquired in childhood, is generally lifelong but progression from the indetermined (asymptomatic) to symptomatic stage occurs in only 30% of cases [2]. A broad pathological spectrum is associated with clinical CD including potentially fatal cardiological and gastrointestinal abnormalities [3]. The relative contributions of parasite and host immunity in driving disease pathology are a matter of continuing debate [4]. Recently, for example, bioluminescent parasite infections in BALB/c mouse models have suggested that heart disease can progress in the absence of detectable local parasite load [5].

It is widely recognized that natural parasitic infections are often comprised of several parasite clones [6]. Malariologists use the term ‘multiplicity of infection’ (MOI) to describe when multiple *Plasmodium sp*. genotypes occur within the same host [7,8]. A similar phenomenon has been observed in *T*. *cruzi* in vectors (e.g. [9]), as well as mammalian reservoir hosts (e.g [10]) and humans hosts (e.g. [11]) using solid phase plating and cell sorting techniques. The occurrence of multi-genotype infections has fundamental implications for host immunity [12], as well as for accurate evaluation of pathogen drug resistance [13], transmission rate, epidemiology and population structure (e.g. [7,11]). The efficiency with which it is possible to sample pathogen clonal diversity from biological samples has soared in recent years with the advent of next generation sequencing. Deep sequencing approaches have long been applied to study the dynamics of HIV anti-viral therapy escape mutations. As a result amplicon sequencing increasingly features in a clinical diagnostic context [14]. *Plasmodium falciparum* MOI can be resolved at merozoite surface protein loci at far greater depths than possible by standard PCR approaches [15]. Furthermore, targeting low copy number antigens in parasite populations via amplicon sequencing can provide important clues to frequency-dependent selection pressures within hosts, between hosts and between host populations [16].

*T*. *cruzi* can persist for several decades within an individual host. Unsurprisingly perhaps, therefore, *T*. *cruzi* shows significant antigenic complexity. *T*. *cruzi* surface proteins are encoded by several large, repetitive gene families that are distributed throughout the parasite genome [17]. Among these gene families the mucins, transialidases, ‘dispersed gene families’ (DGFs), mucin-associated surface proteins (MASPs) and GP63 surface proteases comprise the vast majority of sequences—10–15% of the total genome size [17,18]. Whilst the role of some of the proteins encoded by surface gene families in host cell recognition and invasion is relatively well understood (e.g. the transialidases [19]), the role of others (e.g. the MASPs, DGFs) is not. Furthermore, the role each plays in evading an effective host response remains largely unknown.

The GP63 surface proteases are found in a wide variety of organisms, including parasitic trypanosomatids [20]. In *Leishmania* spp. *GP63* proteases are the most common component of the parasite cell surface with crucial roles in pathogenicity, innate immune evasion, interaction with the host extracellular matrix and ensuring effective phagocytosis by macrophages [21]. In *T*. *brucei* subspp. the role of *GP63* proteins is less well defined, although some protein classes are thought to be involved with variant surface glycoprotein processing between different life cycle stages [22]. In *T*. *cruzi* at least four classes of GP63 gene are recognized [20]. Like many GP63 proteases in *Leishmania* spp., surface expressed *T*. *cruzi* GP63 (TcGP63) genes are anchored to the cell membrane via glycosyl phosphatidylinositol moieties [23,24]. Among these are the TcGP63 Ia & Ib genes (collectively TcGP63I). TcGP63 Ia & Ib encode 78kDa 543 amino acid proteins, are expressed in all life cycle stages and are implicated in the successful invasion of mammalian cells *in vitro* [23,24].

In the current study we target TcGP63I genes as markers of antigenic diversity among three cohorts of Chagas disease patients: two in Cochabamba, Bolivia and one in Goias, Brazil. We also targeted a maxicircle gene for the NADH dehydrogenase subunit 5 to provide basic *T*. *cruzi* genotypic information for each case. Diversity at each of the two *T*. *cruzi* loci within each patient was characterized using a deep amplicon sequencing approach, generating several million sequence reads. Our results shed light on the diversity of parasite DTUs within each patient, as well as the extent to which parasite strains pass between mother and foetus in congenital cases. We were unable to find any evidence that parasite diversity accumulates with age in our study cohorts, or to detect a link between parasite diversity and clinical profile. However, we were able to detect evidence of putative diversifying selection within members of the TcGP63 gene family, suggesting a link between genetic diversity within this gene family and survival in the mammalian host.

## Materials and Methods

### Ethical statement

Ethical permissions were in place at the two centres where human sample collections were made, as well as at the London School of Hygiene and Tropical Medicine (LSHTM). Local ethical approval for the project was given at the Plataforma de Chagas, Facultad de Medicina, UMSS, Cochabamba, Bolivia by the Comite de Bioetica, Facultad de Medcina, UMSS. Local ethical permission for the project was given at the Hospital das Clínicas da Universidade Federal de Goias (UFG), Goias, Brazil by the Comite de Etica em Pesquisa Médica Humana e Animal, protocol number 5659. Ethical approval for sample collection at the LSHTM was given for the overall study, “Comparative epidemiology of genetic lineages of *Trypanosoma cruzi*” protocol number 5483. Samples were collected with written informed consent from the patient and/or their legal guardian.

### Biological sample collection

Parasite isolation protocols were different between centres. At the UMSS, 0.5 mL of whole venous blood was taken from chronic patients and inoculated directly into biphasic blood agar culture. T. cruzi positive samples were minimally repassaged and cryopreserved at log phase (precise repassage history unavailable). For infants, 0.5 mL of chord blood was taken at birth and inoculated into culture. Again, positive samples were cryopreserved at log phase after minimal repassage (precise repassage history unavailable). DNA extractions, using a Roche High-Pure Template Kit, were made directly from the cryopreserved stabilate. At the UFG, 17 mL of whole blood was collected into EDTA, centrifuged for 10 minutes at 1200g at 4°C and the plasma replaced with 8mL Liver Infusion Tryptone (LIT) medium. After a further 10 minutes at 1200g (4°C), the supernatant was again removed. Two mL of packed red blood cells were subsequently transferred to 3 mL of LIT medium and checked periodically for signs of epimastigote growth by light microscopy. Positive cultures were not repassaged. Instead primary cultures were stabilized by the addition of guanidine 6 M-EDTA 0.2 M (Sigma-Aldrich, UK). DNA extractions were made from the full volume using the QIAamp DNA Blood Maxi Kit (Qiagen, UK) according to the manufacturer’s instructions. Among Bolivian strains, DNA concentrations submitted to PCR were standardized after quantitation using a PicoGreen assay. In view of presence of human genetic material in Goias samples, parasite DNA concentrations were standardized to within the same order of magnitude via qPCR as previously described [25]. All samples collected for in this study are listed in Table 1.

**Table 1:** Samples provenance and symptoms.

							TcGp63I Shannon Index	
Code	Sex	Age	Province	Country	Symptoms	ND5 Sequence Type	97% ST	99% ST
**PCC 313**	F	33	Pampa San Miguel, Cochabamba	Bolivia	Cardiopathy	TcIII-VI	0.863833	2.104583
**PCC 221**	F	52	Cercado, Cochabamba	Bolivia	Cardiopathy	TcIII-VI	0.911279	2.757206
**PCC 310**	F	58	Collaj chullpa	Bolivia	Cardiopathy	TcIII-VI	0.80038	2.267772
**PCC 302**	F	55	Oropeza, Chuquisaca	Bolivia	Cardiopathy	TcIII-VI	ND	ND
**PCC 277**	F	50	Pucara Grande, Cochabamba	Bolivia	Cardiopathy	TcIII-VI	0.680276	1.896947
**PCC 460**	M	43	Quillacollo, Cochabamba	Bolivia	Cardiopathy	TcIII-VI	0.64493	1.609727
**PCC 240**	F	36	Pucara grande, Cochabamba	Bolivia	Cardiopathy	TcI	0.19757	0.970659
**PCC 243**	F	19	Chilimarca, Cochabamba	Bolivia	Cardiopathy	TcIII-VI	0.863657	2.235602
**PCC 262**	F	36	Ticti Norte, Cochabamba	Bolivia	Cardiopathy	TcIII-VI	0.602361	2.407113
**PCC 096**	F	58	Cercado-Cochabamba	Bolivia	Asymptomatic	TcIII-VI	ND	ND
**PCC 295**	F	45	Sacaba, Cochabamba	Bolivia	Asymptomatic	TcIII-VI	0.82236	2.641406
**PCC 151**	F	24	Cerro verde, Cochabamba	Bolivia	Asymptomatic	TcIII-VI	0.809111	2.424673
**PCC 253**	M	46	Campero, Cochabamba	Bolivia	Asymptomatic	TcIII-VI	0.765955	2.147924
**PCC 210**	F	40	Cercado, Cochabamba	Bolivia	Asymptomatic	TcIII-VI	0.79258	2.335818
**PCC 263**	M	50	Calicanto, Santa Cruz	Bolivia	Asymptomatic	TcIII-VI	0.654315	1.948427
**PCC 451**	F	32	Uspa Uspa, Cochabamba	Bolivia	Asymptomatic	TcIII-VI	0.715735	2.016351
**PCC 480**	F	46	Cercado, Cochabamba	Bolivia	Asymptomatic	TcIII-VI	0.735419	1.883752
**PCC 481**	F	26	Huayra kasa	Bolivia	Asymptomatic	TcIII-VI	1.12695	3.229099
**PCC 149**	F	20	Cercado, Cochabamba	Bolivia	Asymptomatic	TcIII-VI	0.849494	2.875091
**PCC 502**	F	44	Alto Quer-Queru, Cochabamba	Bolivia	Asymptomatic	TcIII-VI	0.813833	2.570795
**PCC 456**	M	27	Scaba, Cochabamba	Bolivia	Asymptomatic	TcIII-VI	0.529413	1.677289
**PCC 489**	M	46	Quillacollo, Cochabamba	Bolivia	Asymptomatic	TcIII-VI	0.935067	2.67856
**PCC 499**	F	41	Sacaba, Cochabamba	Bolivia	Asymptomatic	TcIII-VI	0.878578	2.402874
**PCC 289**	F	24	Sacaba, Cochabamba	Bolivia	Asymptomatic	TcI	1.0811	ND
**PCC 226**	F	22	Santivañez, Cochabamba	Bolivia	Asymptomatic	TcIII-VI	ND	2.364685
**PCC 255**	F	49	Quillacollo, Cochabamba	Bolivia	Asymptomatic	TcIII-VI	0.703573	1.966296
**PCC 251**	F	37	Sacaba, Cochabamba	Bolivia	Asymptomatic	TcIII-VI	0.774534	2.312599
**6339**	F	58	Sao Luiz MBelos, Goias	Brazil	Cardiopathy	TcII	0.373205	1.409882
**6340**	F	76	Serra do Salitre, Minas Gerais	Brazil	Megaesophagus	TcII	0.19035	1.879923
**6345**	F	67	Formosa, Goias	Brazil	Megacolon, Megaesophagus	TcII/TcI	0.303881	1.574604
**6349**	F	37	Wanderlei, Bahia	Brazil	Megaesophagus	TcII/TcIII-VI	0.841102	1.801845
**6356**	M	65	Itapaci, Goias	Brazil	Cardiopathy, Megacolon, Megaesophagus	TcII	1.168083	2.787444
**6360**	F	40	MaraRosa, Goias	Brazil	Cardiopathy	TcII/TcIII-VI	0.28487	2.332717
**6372**	M	38	Correntina, Bahia	Brazil	Megaesophagus	TcII	0.198327	1.72982
**6373**	F	54	Rubiataba, Goias	Brazil	Megaesophagus	TcII	0.457692	1.929769
**6378**	F	39	Itapaci, Goias	Brazil	Megacolon, megaesophagus	TcII	0.357508	1.259254
**6379** ^z^	M	39	Sao Luiz MBelos, Goias	Brazil	Asymptomatic	TcII/TcIII-VI	ND	ND
**6380**	F	59	Brazabrantes, Goias	Brazil	Asymptomatic	TcII	0.742025	1.786299
**6382**	M	56	Jussara, Goias	Brazil	Megaesophagus	TcII	0.332897	2.104033
**6383**	M	30	Angical, Bahia	Brazil	Asymptomatic	TcII	0.561749	1.653877
**6385**	F	31	SantaMariaVitoria, Bahia	Brazil	Asymptomatic	TcII	0.304854	1.87694
**6386**	M	33	Correntina, Bahia	Brazil	Cardiopathy	TcII/TcIII-VI	0.222146	1.393783
**6387**	F	57	Lagolandia, Goias	Brazil	Cardiopathy (nontypical), Megaoesophagos	TcII	0.07458	1.601748
**6389**	F	24	Cocos, Bahia	Brazil	Asymptomatic	ND	0.025177	1.835489
**6390**	M	32	Cocos, Bahia	Brazil	Cardiopathy	ND	0.037499	1.722559
**6400**	F	47	Correntina, Bahia	Brazil	Asymptomatic	TcII/TcI	0.625396	1.664578
**6401** ^y^	M	30	SantaMariaVitoria, Bahia	Brazil	Megaesophagus	TcII	0.73448	1.570591
**6403**	F	47	Correntina, Bahia	Brazil	Cardiopathy (nontypical) Megaesophagus	TcII/TcI	0.309724	1.136665
**6407**	F	52	Jussara, Goias	Brazil	Cardiopathy (nontypical)	TcII	0.164841	1.86004
**6416** ^x^	M	45	Ceres, Goias	Brazil	Severe cardiopathy	TcII/TcI	0.320845	2.110287
**6423**	M	63	Varzeas, Bahia	Brazil	Megaesophagus	TcII/TcI	0.308932	2.268015
**6425**	M	71	JoaoPinheiro,Minas Gerais	Brazil	Cardiopathy, megaesophagus	ND	0.623522	1.895166
**6445** ^z^	M	40	Sao Luiz MBelos, Goias	Brazil	Cardiopathy (nontypical)	TcII	0.733916	1.707039
**6452** ^x^	M	46	Ceres, Goias	Brazil	Severe cardiopathy	TcII/TcI	0.225277	1.316351
**6453**	M	72	JoaoPinheiro,Minas Gerais	Brazil	Megaesophagus	TcII	0.841132	1.880447
**6536** ^y^	M	30	SantaMariaVitoria, Bahia	Brazil	Megaesophagus	TcII/TcI	0.515696	2.510533
**6548**	F	57	Guiratinga, Mato Grosso	Brazil	Asymptomatic	TcII	ND	ND
**6561**	F	46	Correntina, Bahia	Brazil	Asymptomatic	ND	0.868331	1.82152
**6563**	F	58	Sao Domingos, Goias	Brazil	Cardiopathy (non-typical)	TcII	1.049223	2.262427
**6569**	M	65	Anapolis, Goias	Brazil	Megaesophagus	TcII/TcIII-VI	0.615555	1.578375
**6571**	F	69	Uruana, Goias	Brazil	Cardiopathy, megaesophagus	ND	0.366073	2.659534
**6574**	F	39	SantaMaria da Vitoria, Bahia	Brazil	Megaesophagus	ND	0.846975	1.682228
**6577**	F	63	Damolandia, Goias	Brazil	Cardiopathy, Megacolon, Megaesophagus	ND	0.258461	1.892257
**6581**	M	55	Rubiataba, Goias	Brazil	Cardiopathy	TcII	0.820042	2.019464
**6582**	M	45	MorroChapeu, Bahia	Brazil	Cardiopathy, megacolon, megaesophagus	TcII/TcIII-VI	1.079722	1.940343
**6588**	M	51	Luziania, Goias	Brazil	Cardiopathy	TcII	0.197856	0.719487
**6590**	M	49	Itaberai, Goias	Brazil	Severe cardiopathy	TcII/TcIII-VI	0.32446	2.4802
**6597**	F	56	Almas, TO	Brazil	Megaesophagus, megacolon	TcII/TcI	0.640673	1.910199
**6603**	F	36	SantaMariaVitoria, Bahia	Brazil	Cardiopathy	TcII	0.403289	1.987725
**6687**	F	49	Arapua, Minas Gerais	Brazil	Cardiopathy, megaesophagus	TcII/TcI	0.390482	1.752364
**6718** ^a^	F	28	Sao Luiz MBelos, Goias	Brazil	Cardiopathy	TcII/TcI	0.281918	1.826416
**6720** ^a^	F	1	Sao Luiz MBelos, Goias	Brazil	Acute phase	TcII/TcI	0.224137	1.67497
**CIUF 45 (B1)**	ND	<1	Cochabamba	Bolivia	Congenital	TcIII-VI	ND	1.679934
**CIUF 63 (M1)**	F	18	Cochabamba	Bolivia	Congenital	TcIII-VI	ND	1.582106
**CIUF 91 (B10)**	ND	<1	Cochabamba	Bolivia	Congenital	TcIII-VI	ND	1.948954
**CIUF 84 (M10)**	F	35	Cochabamba	Bolivia	Congenital	TcIII-VI	ND	2.247228
**CIUF24 (B2)**	ND	<1	Alto Cochabamba	Bolivia	Congenital	TcIII-VI	ND	1.390874
**CIUF31 (M2)**	F	20	Alto Cochabamba	Bolivia	Congenital	TcIII-VI	ND	1.662247
**CIUF40 (B3)**	ND	<1	Sucre	Bolivia	Congenital	TcIII-VI	ND	1.621565
**CIUF25 (M3)**	F	19	Sucre	Bolivia	Congenital	TcIII-VI	ND	1.231294
**CIUF 42 (B4)**	ND	<1	Cochabamba	Bolivia	Congenital	TcIII-VI	ND	1.886761
**CIUF26 (M4)**	F	21	Cochabamba	Bolivia	Congenital	TcIII-VI	ND	1.914621
**CIUF65 (B5)**	ND	<1	Cochabamba	Bolivia	Congenital	TcIII-VI/TcI	ND	1.578691
**CIUF75 (M5)**	F	17	Cochabamba	Bolivia	Congenital	TcIII-VI/TcI	ND	1.576942
**CIUF 105 (B6)**	ND	<1	Quillacollo, Cochabamba	Bolivia	Congenital	TcIII-VI	ND	2.330751
**CIUF 104 (M6)**	F	17	Quillacollo, Cochabamba	Bolivia	Congenital	TcIII-VI	ND	2.082838
**CIUF 53 (B7)**	ND	<1	Sucre	Bolivia	Congenital	TcIII-VI	ND	1.6921
**CIUF 76 (M7)**	F	27	Sucre	Bolivia	Congenital	TcIII-VI	ND	2.683029
**CIUF 35 (B8)**	ND	<1	Chimba, Cochabamba	Bolivia	Congenital	TcIII-VI	ND	1.992589
**CIUF 93 (M8)**	F	ND	Chimba, Cochabamba	Bolivia	Congenital	TcIII-VI	ND	1.653714
**CIUF 98 (B9)**	ND	<1	Vinto, Cochabamba	Bolivia	Congenital	TcIII-VI	ND	1.983814
**CIUF 109 (M9)**	F	18	Vinto, Cochabamba	Bolivia	Congenital	TcIII-VI	ND	1.716494

^a^ Samples from Goias congenital case

^x^ Samples from the same patient taken >12 months apart

^y^ Samples from the same patient taken < 6 months apart

^z^ Samples taken from the same patient >12 months apart

### Epidemiological and clinical observations

The two areas studied have dissimilar histories in terms of Chagas disease transmission intensity. Vector-borne *T*. *cruzi* transmission in Goias and its surrounding states (where samples were collected—Table 1) was interrupted approximately 20 years before the sampling detailed in this study [26,27]. In the sub-Andean semi-arid valleys of Cochabamba and its environs, however, vector-borne domestic transmission is still a likely source of new infections, albeit at a reduced rate since intensive spraying campaigns in the mid 2000s [28]. Clinical data collected in this study were categorised simply into symptomatic and asymptomatic classes for statistical tests in view of samples sizes. Sub-categories within symptoms were defined as 1) Cardiopathy (including any electrocardiographic and/ or echocardiographic abnormalities, X-ray with cardiac enlargement. Patients with atypical cardiac abnormalities i.e. those not exclusively associated with Chagas disease, were included in the symptomatic class in the context of this study.) 2) Megaesophagous (including achalasia and barium swallow abnormalities) 3) Megacolon (constipation associated with dilation as by barium enema) and 4) Normal (no symptoms or signs on examination and a normal electrocardiogram) (Table 1)

### Primer design, PCR conditions, amplicon sequencing and controls

Degenerate primers for a 450bp fragment of the maxi-circle NADH dehydrogenase 5 were designed as described in Messenger et al. 2012 [29]. Degenerate primer design for the TcGP63I family surface proteases (including Ia and Ib sublaclasses) [24] was achieved by reference to sequences retrieved from EuPathDB for Esmeraldo (TcII), CL Brener (TcVI), Silvio (TcI) and JR (TcI) (http://eupathdb.org/). Primer biding site positions in relation to TcGP63I putative functional domains are displayed in S1 Fig. Homologs were identified by BLAST similarity to a complete TcGP63I sequence (bit score (S) ≥ 1000). Alignments of resulting sequences were made in MUSCLE [30] and primers were designed manually to target a variable region within and between individual strains with a final size of 450bp. ND5b primer sequences were ND5b_F ARAGTACACAGTTTGGRYTRCAYA; ND5b_R CTTGCYAARATACAACCACAA. The final TcGP63 primers were TcGP63_F RGAACCGATGTCATGGGGCAA and TcGP63_R CCAGYTGGTGTAATRCTGCYGCC. Amplification was undertaken using the Fluidigm platform and a reduction of the manufacturer’s recommended number of cycles to total of 26 was made in an attempt to minimise PCR amplification bias. Thus, the manufacturer’s recommended conditions were adapted to the following protocol: one cycle of 50°C for 2 minutes, 70°C for 20 minutes, and 95°C for 10 minutes; six cycles of 95°C for 15 seconds, 60°C for 30 seconds, 72°C for 60 seconds; two cycles of 95°C for 15 seconds, 80°C for 30 seconds, 60°C for 30 seconds and 72°C for 60s; five cycles of 95°C for 15 seconds, 60°C for 30 seconds, 72°C for 60 seconds; two cycles of 95°C for 15 seconds, 80°C for 30 seconds, 60°C for 30 seconds and 72°C for 60 seconds; five cycles of 95°C for 15 seconds, 60°C for 30 seconds, 72°C for 60 seconds, and finally five cycles of 95°C for 15 seconds, 80°C for 30 seconds, 60°C for 30 seconds and 72°C for 60 seconds. Amplifications were performed using the FastStart High Fidelity PCR System (Roche). Three PCR reactions were pooled per sample prior to sequencing in an attempt to further reduce amplification biases [31]. Equimolar concentrations of ND5 and TcGP63I amplicons from 96 DNA samples were multiplexed on Illumina runs using dual index sequence tags (Illumina Inc). Sequencing was undertaken using a MiSeq platform using a 2 x 250 bp (Reagent Kit version 2) according to the manufacturer’s protocol. In addition to the clinical samples, we included a dilution series of control samples. The controls comprised artificially mixes of DTUs I-VI genomic DNA at equimolar concentrations. At the ND5 locus, comparison between the expected DTU abundance ratios and diversity of artificial control mixes and that defined via amplicon sequencing was made (S2 Fig.).

### Amplicon sequence data analysis

De-multiplexed paired-end sequences were submitted to quality control and trimming in Sickle [32] and mate pairs trimmed in FASTX Toolkit (http://hannonlab.cshl.edu/fastx_toolkit/). ND5, TcGP63 and contaminating sequences were then sorted against a reference using BOWTIE2 [33]. Individual paired reads were found to be overlapping in only a minority of cases. Thus we chose to proceed with analysis of a sequence fragment with a truncated central section for both targets. Further sequence manipulations were undertaken using FASTX Toolkit and custom *awk* scripts to parse files and concatenate each mate pair into a single sequence for downstream analysis. MUSCLE [30] was used for alignment of amplicon sequences in each patient sample. Next, analysis was undertaken in the Mothur software package [34] for the elimination of putative PCR chimeras and individual sequence clustering. The Shannon index of diversity was calculated at the intra-patient level based on sequence types (STs) defined at 97% and 99% identity in Mothur [34]. Comparisons of Shannon diversity were made between patients in each cohort (Bolivia chronic, Bolivia congenital, Goias chronic) via analyses of covariance and linear regression in the R package (http://CRAN.R-project.org). TcGP63I sequence datasets for patients from each cohort were then merged and analyses conducted using 97% and 99% STs defined with UPARSE [35] across patients. Weighted UniFrac distances between TcGP63I STs among samples were generated and subsequently clustered via a principal coordinates analysis in QIIME [36]. Significance of association between UniFrac clustering, disease status and age was tested in the vegan package in R [37]. Estimates of diversifying selection among TcGP63I STs were made in KaKs Calculator [38] using Yang and Neilson’s 2000 approximate method [39] and tested for significance using a Fisher’s exact test. Prior to selection calculations, sequences were clustered into 99% identity STs and singletons excluded in an attempt to exclude SNPs introduced as PCR artefacts. To test for diversifying selection across putative TcGP63I gene families (TcGP63Ia & Ib—97% cut-off as defined by Cuevas and colleagues [24]), 99% identity STs from each patient cohort were pooled (Table 2). To test for selection within TcGP63I gene families, STs within each 97% category (corresponding to TcGP63Ia & b respectively) were examined separately per cohort (Table 2). Amplicon sequences analysed in this study are available in the data appendix in supplementary information (S1 Appendix).

**Table 2:** Yang and Neilson estimates for positive selection within and among abundant 97% STs identified in this study.

Population / ST	Infecting strain	Sequences in cluster^a^	Method	Ka	Ks	Ka/Ks	P-Value^b^	S-Sites	N-Sites	Substitutions	S-Substitutions	N-Substitutions
**Goias**	TcII	357227 (271)	YN	*0*.*06*	*0*.*07*	*0*.*8354*	*0*.*0000*	14910	37539.0	3061	974.0	2087.0
**Goias ST1**	TcII	236805 (149)	YN	**7.45**	**2.82**	**2.6436**	**0.0000**	6628.46	20575.5	20094	4528.3	15565.7
**Goias ST2**	TcII	96274 (82)	YN	**7.04**	**1.11**	**6.3415**	**0.0000**	4112.28	12000.7	11713	2328.3	9384.7
**Goias ST4**	TcII	9981 (19)	YN	0.02	0.05	0.4151	0.0000	978.551	2543.5	102	48.4	53.6
**Bolivia**	TcV/TcI	59431 (86)	YN	0.06	0.08	0.7515	*0*.*0002*	4333.81	10471.2	904	314.8	589.2
**Bolivia ST1**	TcV	38455 (36)	YN	0.02	0.03	0.7876	0.1600	2092.15	5077.9	182	62.0	120.0
**Bolivia ST2**	TcV	12676 (24)	YN	0.03	0.03	0.7868	0.2290	1208.92	3471.1	134	41.0	93.0
**Bolivia ST3**	TcI	3448 (13)	YN	**5.57**	**1.98**	**2.8059**	**0.0000**	582.981	1679.0	1739	402.5	1336.5
**Bolivia ST4**	TcI	242 (3)	YN	4.48	3.61	1.2422	0.3484	138.391	392.6	410	102.9	307.1

^a^ Numbers in brackets represent the number of 99% STs define within each cluster from which estimates were generated.

^b^ P values are give for Fisher’s exact tests for deviation from the neutral expectation of Ka/Ks = 0.

## Results

### Sequence yields and discrete typing unit (DTU) designations

**Fig 1:**
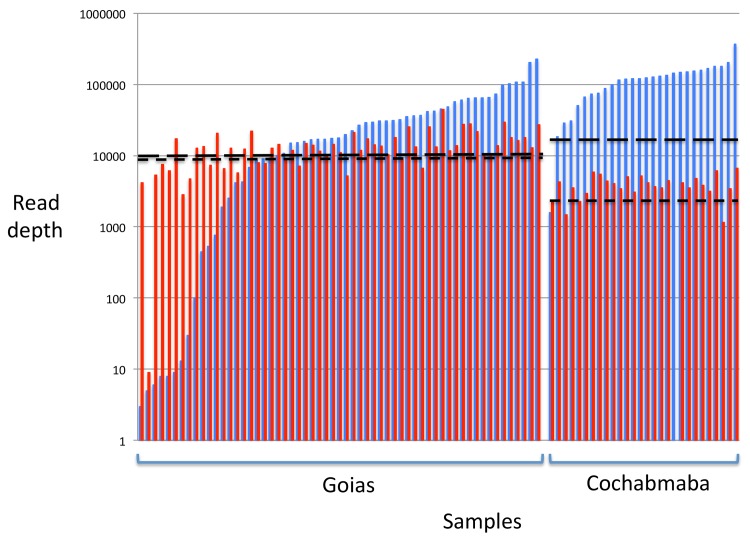
Read depths by sample and locus for Goias and Cochabamba chronic patient cohorts after quality filtering. Read depths generated on the Illumina MiSeq platform were standardized across samples prior to analysis. Inclusion thresholds for TcGP63 (Goias—10,000; Cochabamba—3000; wide dash line; red bars) and ND5 (Goais—10,000; Cochabamba—30,000; thin dash line; blue bars) are shown for each population.

After quality filtering, trimming, decontamination and removal of unpaired reads, 6,736,749 reads were assigned to the ND5 mitochondrial marker and 871,855 to TcGP63I marker across the 92 clinical samples, perhaps reflecting higher copy number in the former than the latter. After trimming, the overlap between individual mate pairs was marginally too short to be assembled into a single read. Thus paired reads were first aligned against a full-length reference fragment and the central portion excised to remove any gaps and ensure correct alignment. Sequence depth thresholds per sample for inclusion were set for each dataset (Goias—ND5 & TcGP63–10,000; Cochabamba—ND5: 30,000; TcGP63 10,000; see Fig. 1). Reads from samples in excess of this threshold were discarded and samples with read counts below this threshold excluded. Our aim in setting the threshold was: 1) To include as many samples as possible while maintaining a good depth of coverage; 2) To standardise sampling intensity across individuals and thus facilitate comparisons between them.

The ND5 mitochondrial target was sequenced to provide DTU I-VI identification of parasites circulating within and among patients by comparison to existing data [29]. However, with reference to the results from the control samples—and due the necessary truncation of the sequence fragment—only three groups could be reliably distinguished, corresponding to the three major *T*. *cruzi* maxicircle sequence classes [40]. The three groups corresponded to TcI, TcII and TcIII-VI respectively. Furthermore, in reference to the control mixes, we found evidence that amplification bias dramatically skewed the recovery of sequence types (STs) towards the TcIII-VI group. Some skew is expected, as these four DTUs (TcIII-VI) share the same maxicircle sequence class, and this class is thus more abundant in the control mix. However, TcI and TcII—which should have in theory been present as 16% (1/6) of all sequences in the controls respectively—were in fact present (on average) at only 2.9% and 0.03% among the four samples where all three STs were recovered (S2 Fig.). Amplicon sequencing from the two most concentrated controls (57 ng/uL and 125 ng/uL genomic DNA respectively) resulted in poor sequence yields and a failure to recover all three STs.

**Fig 2:**
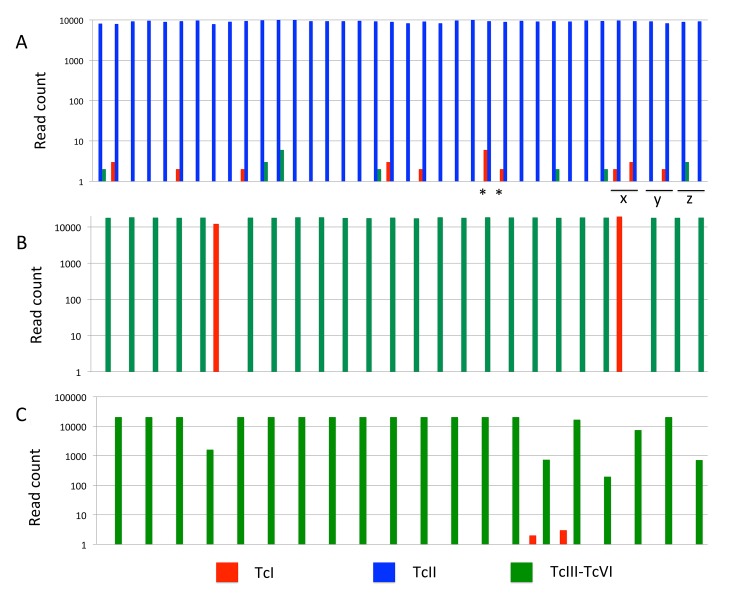
Bar plot showing sequence type identity and abundance defined at 97% similarity for the ND5 locus across all samples. A—Goias cohort chronic/intermediate cases; B—Cochabamba chronic/intermediate cases; C—Cochabamba congenital cases. Y axes show log transformed abundance (read counts). X axes show clustered bars for individual samples. Sequence type identities are given in the legend. Stars denote congenital pair from Goias. Labels x (6416 / 6452), y (6401 / 6536) and z (6379 / 6445) sample pairs from the same patient at different time points (see Table 1).

Unsurprisingly perhaps in the light of the control data, most clinical samples were dominated by sequences from a single group, with minor contributions from others (Fig. 2). Indeed sequences recovered from many strains were monomorphic at the 97% identity level—especially in Cochabamba. As such, comparisons based on ND5 are necessarily descriptive and meaningful alpha (within sample) and beta (between sample) diversity statistics were not calculated. Fig. 2 shows the distribution of DTUs among samples as defined by the ND5 locus. Most Cochabamba chronic cases samples were assigned to a single sequence within the TcIII-VI group (likely to be TcV, as we defined with standard genotyping assays [41] with the exception to two TcI cases—PCC 240 and PCC 289 (Fig. 2, Panel B). Sequence-type diversity in Goias was considerably higher (Fig. 2, Panel A). In this case the TcII group, rather than the TcIII-VI group, predominated. Unlike in Bolivia, sequences from other groups were present alongside TcII in multiple patients but at frequencies two orders of magnitude lower. Congenital pairs that originated from Cochabamba resembled chronic cases from the same region in their DTU composition (TcIII-VI group predominant, Fig. 2, Panel C). Strikingly, mother/child pair CIUF65 (B5) and CIUF75 (M5) share similar mixed infection profiles (TcI/ TcIII-VI) at similar relative abundances (*c*.1:1000), consistent with the minor to major genotype abundance ratios observed in Goias. The same is also true for the Goias congenital pair (Fig. 1) which both showed TcII/TcI mixes. Finally, sequential isolates taken from the same Goias chronic patient at different time points suggest that minor abundance genotypes are not always consistently detectable in the blood (Fig. 2): TcI is absent at first sampling of patient *y*, but present at the second sampling. For patient *z*, the TcIII-VI genotype is only present in the first of the two sample points. For both Cochabamba and Goias, reference to the control data suggests that ‘minor genotypes’ could be substantially more abundant in the patients than the amplicon sequence data suggest.

### TcGP63I surface protease alpha diversity among clinical and congenital cases

Alpha diversity measurements aim to summarise the diversity of species (in this case STs), within an ecological unit (in this case a host). We summarized the number of STs and their relative abundance in each of our samples, using the Shannon Index (SI) [42]. Among non-congenital cases, our aim was to evaluate possible associations between TcGP63I antigenic diversity and several epidemiological and clinical parameters—age, sex and disease status. We used analyses of covariance (ANCOVA) to test for the effect of these parameters on intra-host antigenic diversity (STs defined both at 97% and 99% for comparison), combining continuous (age) and categorical (sex, clinical forms) data. In Cochabamba, regardless of the order in which parameters were included as factors in the model, there was no evidence for a main effect of age, sex or symptoms on alpha diversity (SI) at either ST divergence level (*97% ST* Age: p = 0.734; Sex: p = 0.298; clinical form: p = 0.136. *99% ST*—Age: p = 0.854; Sex: p = 0.169; clinical form = 0.0988). Similarly, ANCOVAs were non-significant for an association between the SI and age, sex or symptoms in Goias (*97% ST*—Age: p = 0.382; Sex: p = 0.535; clinical form: p = 0.486. *99% ST*—Age: p = 0.319; Sex: p = 0.696; clinical form: p = 0.697). Finally, we undertook linear regressions of SI with age in each population. As one might expect from previous ANCOVAs, no significant correlation was detected (Goias *R* ^*2*^ = 0.0233, *p* = 0.340 (97% ST); *R* ^*2*^ = 0.0256, *p* = 0.3049 (99% ST) Cochabamba *R* ^*2*^ = 0.0287, *p* = 0.429 (97% ST); *R* ^*2*^ = 0.0230 *p* = 0.479(99% ST)).

**Fig 3:**
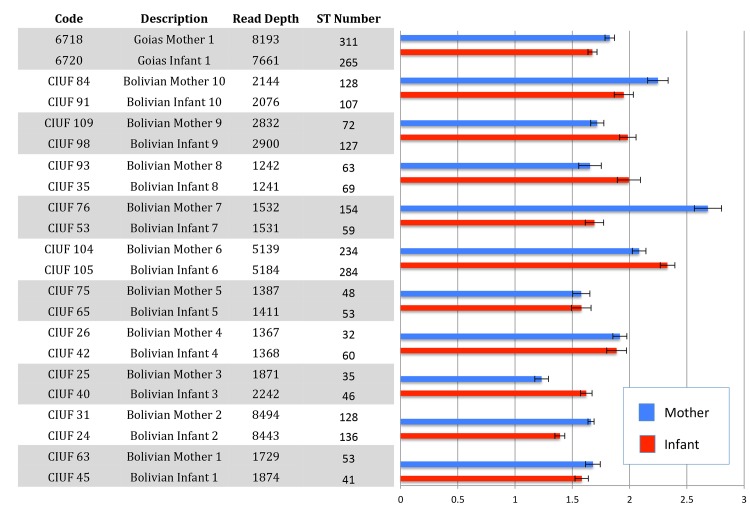
Alpha diversity indices for TcGP63I amplicon diversity derived from pairs of congenital Chagas disease cases. Diversity indices were derived from STs defined at 99% sequence similarity. Bar plot and associated *x*-axis on the right hand side shows the Shannon diversity index calculated in Mothur [34], with error bars defining upper and lower 95% confidence intervals.

Congenital comparisons were made pairwise between mother and infant at 99% ST similarity. In addition to the ten matched isolate pairs from Cochabamba, a single pair from Goias was also included (6718 & 6720) in the comparisons. The results of the alpha diversity comparisons are shown in Fig. 3, and read depths were balanced between samples. In terms of the absolute number of STs identified, infants exceeded mothers in most instances (pairs 2, 3, 4, 5, 6, 8 & 9). In the remaining cases however (4/11), the number of antigenic sequence types was greater in the mother. Shannon diversity index comparisons between mothers and infants, which also takes ST abundance into account, suggested that some differences (e.g. pairs 4, 5 &6) might be marginal (Fig. 3).

### TcGP63I ST distributions among clinical and congenital CD patients

**Fig 4:**
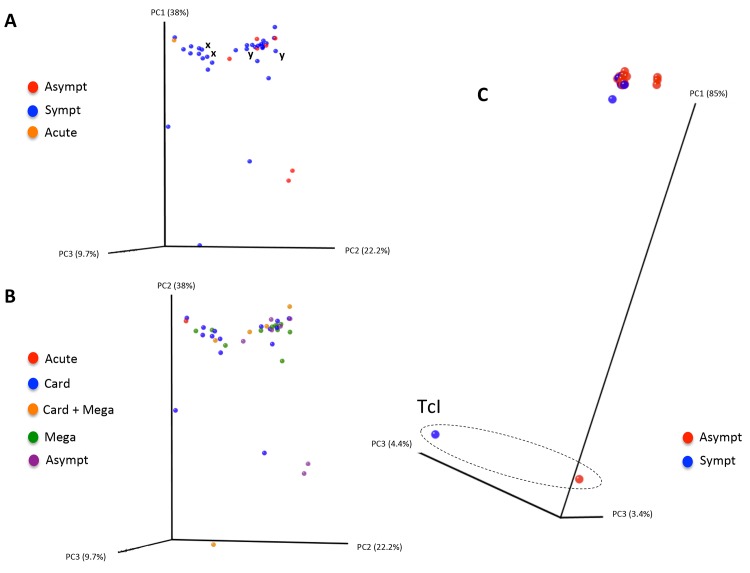
Principal coordinates analysis of sequence diversity between chronic Chagas Disease patient TcGP63I antigenic repertoires. Genetic distances are based on a weighted unifrac metric. Plot A shows diversity comparisons among Go-as asymptomatic (asympt) and symptomatic (sympt) clinical cases, as well as one acute case. Plot B shows Goias cases with symptoms categorised as acute, card (cardiopathy), card + mega (cardiopathy as well as megacolon and / or megaesophagous), mega (megacolon and / or megaesophagous) or asympt (asymptomatic). Plot C shows comparisons among Cochabamba clinical cases (not including congenital cases) classified as either asymptomatic (asympt) and symptomatic (sympt). The dashed circle on plot C indicates samples unambiguously defined as TcI at the ND5 locus. Pairs of sequential isolates from the same patient are labelled x and y respectively.

Individual sample sequence datasets within each of the different study cohorts (Cochabamba congenital, Cochabamba non-congenital and Goias) were merged to facilitate analysis of the distribution of antigen 99% STs among individuals (i.e. beta-diversity comparisons). Pairwise weighted Unifrac distances were calculated within cohorts of chronic cases from Cochabamba and Goias to examine whether the sequence diversity of the TcGP63I antigenic repertoire present in each patient could be associated with disease outcome. Principal coordinate analyses of the resulting matrices are displayed in Fig. 4. Among cases from Goias, repertoires varied considerably among cases, with several outliers. However, repertoires from symptomatic and asymptomatic cases were broadly overlapping in terms of sequence identity, and no clustering was noted among different symptom classes either (Fig. 4, Plot B). Permutational multivariate analysis confirmed the absence of a link between ST clustering and symptoms as well as symptom classes (*p* = 0.77 & 0.74 respectively). However, ST clustering and age were weakly associated (*p* = 0.049), consistent perhaps with exposure of individuals among different age groups to different circulating parasite genotypes at their time of infection. TcGP63I read yields permitted comparisons for only two pairs of sequential isolates from the sample patients—*x* and *y* (see Table 1)—both of which showed closely clustering, although non-identical, profiles. TcGP63I diversity between Cochabamba chronic cases was arguably lower, with the exception of two outliers unambiguously identified as TcI with reference to the ND5 locus (all others were classified as TcIII-VI—likely TcV). Again, however, symptomatic and asymptomatic cases were broadly overlapping.

**Fig 5:**
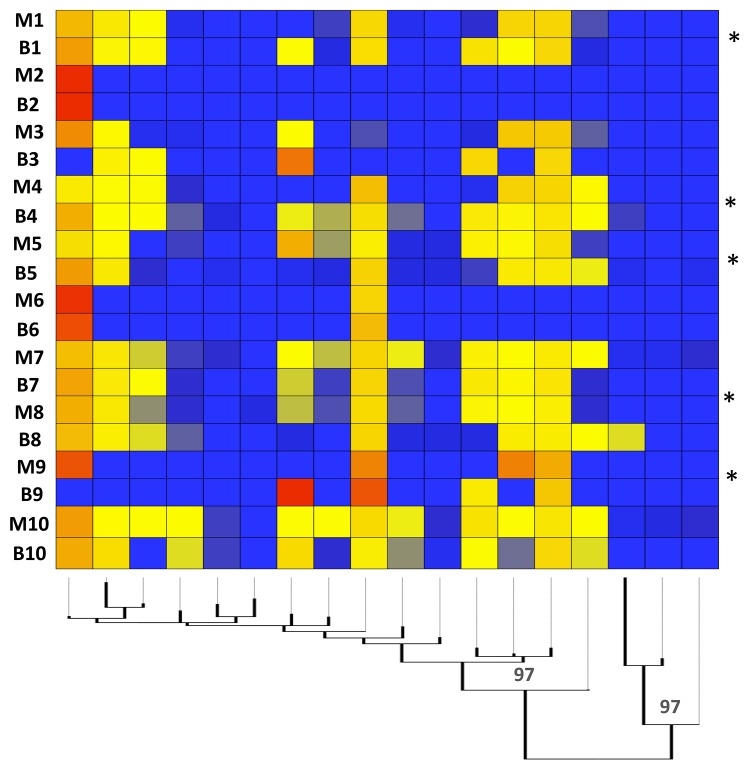
Heatmap comparing the TcGP63I antigenic repertoires of mother and infant congenital pairs. Pairs are indicated down the left hand side of the image (y axis). The mid-point rooted maximum likelihood tree on the x axis describes relationships among the 99% similarity sequence types (STs) identified in UPRASE [35] and was generated in Topali under equal-frequency transversion model, allowing gamma distributed weights across sites [68]. Values on dendrogram notes indicate % bootstrap support. Starred congenital pairs are those where STs are present in the infant but not in the mother.

Sequence type profile comparisons among Cochabamba congenital cases were made for 99% STs and are displayed in heatmap format in Fig. 5. There are two key features of interest. The first is that profiles in mother an infant can match very closely (e.g. pairs 2&6). The second is that novel STs were present in the infant sample with respect to the mother in half of the cases. Indeed, in pair 9, the infant profile was radically different to that of the mother.

### Population-level Ka/Ks ratios within and between TcGP63I gene family members

Trimmed TcGP63 reads, pre-filtered for quality and PCR errors, were pooled within each study site (Bolivia, Goias). To further reduce minority SNPs and PCR errors, STs were defined at 99% with each site in UPARSE [35]. *Ka/Ks* ratio estimates within each study area indicated a significant excess of synonymous mutations among STs (Goias = 0.8354, Bolivia = 0.7515) averaged across sites (Table 2). However, when calculations were based on diversity present among well represented STs of each gene family member (TcGP63Ia and TcGP63Ib, 97% cut-off [24]) a powerful and significant excess of non-synonymous substitutions was noted within each study area (*Ka/Ks*, Goias, ST1 = 2.6436, ST4 = 6.3415; Bolivia ST3 = 2.8059; Table 2). Again, calculations were based not on individual sequences, but rather 99% STs within predefined 97% clusters. The position of the 97% STs in question is shown in the tree in S3 Fig., with clear similarity between those clusters under apparent diversifying selection (Goias ST1 & 2, and Bolivia ST3) with TcGP63Ia and TcGP63Ib references respectively [24].

## Discussion

In this study our aim was to collect a cohort of *T*. *cruzi* samples from clinical CD cases, representative of different endemic regions and of different ages and disease presentations, to explore links between CD epidemiology and multiplicity of infection. To provide a robust, sensitive and quantifiable means of assessing intra-host parasite diversity we first implemented standardized parasite isolation (and enrichment) strategies within each study cohort. Latterly, we developed an amplicon sequencing approach to profile parasite diversity within each patient. Given the relatively short (400–500bp) read lengths generated by next generation sequencing platforms (at the time of experimentation), we chose a rapidly evolving maxicircle gene (ND5) in an attempt to resolve DTU level diversity ([29]). Current multilocus nuclear targets are generally too long (500bp+) to meet our selection criteria [43]). To explore antigenic diversity, we chose a putatively low (5–10) copy number gene family member TcGP63I, expressed on the parasite surface during the amastigote and trypomastigote lifecycle stage and thus exposed to the human immune system [24]. Given that both ND5 and TcGP63I are present as several copies per parasite genome (and potentially show inter-strain copy number variation e.g. [44]), one cannot presume a 1:1 relationship between ST and parasite individual, even if we were able to account for the PCR amplification bias we detected. The identification of a genetically, variable, single copy, surface expressed antigen locus is a major challenge in *T*. *cruzi*—antigen genes are by their nature highly repetitive [17,18]. TcGP63I, with its relatively low copy number represents the closest currently available fit, and, as we have shown, provides a useful target for revealing intra-host antigenic diversity. Merozoite surface proteins (MSP) 1 and 2 have traditionally provided useful targets for detecting MOI in *P*. *falciparum* (e.g. [45,46]. Furthermore, amplicon sequencing of the MSP locus has been successfully proven to reveal as many as six-fold more variants than traditional PCR-based approaches [15].

The substantial historical interest in defining MOI among *P*. *falciparum* owes itself to the strong correlation between MOI and rate of parasite transmission [47]. As such, fluctuations in transmission intensity can be tracked to evaluate the efficiency of vector eradication campaigns, drug treatments, the introduction of insecticide-treated nets etc—without the need to directly estimate the entomological inoculation rate. Evaluation of CD transmission intensity has its own challenges. The presence of infected individuals, triatomine vectors in domestic buildings, incrimination of vectors via human blood meal identification (e.g. [48]) can all help to build the overall picture. However, parasite transmission is likely to occur in only a tiny proportion of blood meals [49,50], and vector efficiency is thought to vary considerably between triatomine species [51]—thus the presence of vectors is no guarantee of transmission. Infection with *T*. *cruzi* is lifelong, thus positive patient serology is not a reliable indicator of active parasite transmission either. Traditionally, active *T*. *cruzi* transmission has been implied from positive serology among younger age classes. Especially in hyperendemic areas of Bolivia, Paraguay and Argentina the proportion of seroprevalent individuals increases with age [52,53]. MOI in *T*. *cruzi* patients should follow a similar trend given a stable force of infection. Furthermore MOI comparisons between disease foci could, controlling for age, facilitate an appreciation of relative transmission intensities—a useful tool for those who wish to track the efficacy of interventions. In the current study, however, we were unable to identify a correlation between MOI and age, even once patient sex and clinical form had been corrected for. Our inability to validate this fundamental prediction has many possible causes. First, patients in each cohort originate from different communities within each study area (Table 1). Micro-geographic variation in *T*. *cruzi* genetic diversity is commonly observed (e.g. [11,54,55], and the same is likely to be true for infection intensity. Thus, if patients from different sites share dissimilar histories in the intensity and diversity of exposure to *T*. *cruzi* clones, comparisons between them are difficult to make. Secondly, the relationship between MOI and age is not necessarily linear. If a degree of cross-genotype immunity accumulates with exposure, one might expect a slower increase in intra-host antigenic diversity in older age groups. However, this was not the case in our dataset and neither a linear, nor a unimodal relationship could be established.

Amplicon sequencing approaches to the study of transmission patterns in human parasites have so far been restricted to those species that replicate and reach high parasitemias in peripheral blood (i.e. *T*. *brucei* [56] and *P*. *falciparum* [13,15]). *T*. *cruzi* trypomastigote circulating parasitemias, as measured by qPCR, are thought to vary considerably between acute (400 parasites/ml), newborn (150–12000 parasites/ml) and chronic (3–16 parasites/ml) cases [25,57]. Nonetheless, they remain several orders of magnitude lower than those that occur during *T*. *brucei* or *P*. *falciparum* infections. Low circulating *T*. *cruzi* parasitemia presents major problems to studies that aim to achieve molecular diagnosis of CD in chronic cases and ours is no exception. One problem is that much of the parasite diversity present in the host is likely to be sequestered in the tissues at any give time [58], as our sequential samples from Goias also suggest. Thus blood stage parasite genetic diversity may be a poor representation of that actually present in the host. Another confounder is culture bias, by which differential growth of clones in culture, as well as loss of clonal diversity during repassage can both influence diversity estimates. Attempts to generate amplicon sequence data directly from clinical blood samples would likely to be thwarted by low circulating parasitemia [25, 56]. Instead we elected to enrich for parasite DNA via culture—in Goias without further repassage, but in Bolivia with at least one repassage before cryopreservation. Low circulating parasitemia in Chagas patients also means it is possible that amplicon-sequencing strategies might rapidly ‘bottom out,’ if few parasites are present within a sample. In our dataset, for example, at the ND5 locus, minority DTUs at 97% divergence can be present as a proportion of < 1 in 1000 (Fig. 1), with the implication that several thousand parasites must be present in the sample. In both Goias and Bolivia matched instances occurred in congenital cases where TcI exists in mother and infant as the minor DTU at similar relative abundance (i.e. 1 in 1000, Fig. 1). It is highly unlikely that these data directly reflect chronic CD parasitemia levels. Instead, with reference to the data we obtained from the controls, PCR amplification bias is a more likely source of unrealistic major to minor genotype ratios. As such, the fourfold over-representation of a ST in the original sample, for example, can result in 100–1000 fold over-representation after PCR. However, while the relative abundance of sequence types recovered using the amplicon approach may be an inaccurate reflection of those present for both ND5 and TcGP63, similar profiles between mother and infant suggests that this bias is likely to be consistent across samples. Thus comparisons between samples are still valid. Furthermore for ND5 at least it seems that *T*. *cruzi* frequently exchanges mitochondrial (maxicircle) genomes with little apparent evidence of nuclear exchange [11,29]. Fusion of maxicircle genomes occurs transiently during *T*. *brucei* genetic exchange events [59], and may also do so in *T*. *cruzi*. Even though standard maxicircle genotyping of progeny only ever reveals a single parent in both species, it is possible that heterologous maxicircle sequences may persist at low abundance in parasite clones. Such a phenomenon could explain the DTU sequence type ratios observed, and this study is the first to sequence a maxicircle gene to this depth.

There is general consensus in the literature is that the likelihood of congenital CD transmission is not strongly influenced by the genotype of the parasite infecting the mother [60–62]. Nonetheless, the majority of cases are reported in the Southern Cone region of South America, providing a circumstantial link with major human-associated *T*. *cruzi* genotypes TcV TcII, and TcVI. In this study, in the one mixed infection we found, major and minor DTUs (TcVI / TcI) detected in the mother at the ND5 locus were recovered from the infant in similar proportions. TcGP63I beta diversity comparisons of STs defined at 99% showed substantial sharing of between mother and infant (Fig. 5). However, both beta diversity comparisons (Fig. 5) and total ST diversity (alpha) comparisons (Fig. 3) at 99% indicate that while maternal diversity sometimes exceeds that of the infant (explicable perhaps by sequestration in the mother and selective or stochastic trans-placental transfer), the reverse is frequently true. The occurrence of STs in the infant, not present in the mother, has several possible explanations. The infants sampled in this study were neonates, thus superinfection can be ruled out as a source of further parasite clonal diversity. A recent study of infected neonates in Argentina estimated mean infant parasitemia at 1,789 parasites/ml via qPCR—far in excess of that one might expect in the mother [57]. Thus the parasite sample size discrepancy between mother and infant perhaps explains the unexpected levels of diversity in the infant. Even though the TcGP63I gene family is apparently under intense diversifying selection, it seems unlikely that point mutation could generate novel variants over such a short time scale to explain genetic diversity in the infant. Structural variants and homologous recombination are a potential source of diversity, although most, if not all of recombinants should have been excluded in the quality filtering stages, and would be hard to distinguish from PCR chimeras in any case.

Many important *T*. *cruzi* surface genes belong to large, recently expanded paralogous multigene families [17]. The abundance of these gene copies highlights their likely adaptive significance in terms of infectivity and host immune evasion, especially because trypansomatids exert so little control of gene expression at the level of transcription [63]. In *Leishmania major*, for example, it has been recently shown that gene amplification may rapidly duplicate segments of the genome in response to environmental stress [64]. As well as expansion, adaptive change is also likely to occur at the amino acid level among members of paralogous gene families, as has been suggested for *T*. *brucei* [65]. Despite the relatively small size of the TcGP63I gene family, the amplicon sequencing approach we employed allowed us to explore selection at the level of the gene within the population, i.e. within and between parasite genomes within and between hosts at the population level. Highly elevated non-synonymous substitutions suggest intense diversifying selection within TcGP63Ia and TcGP63Ia STs respectively for those assigned to TcII or TcI. STs from patients infected with TcIII-TcVI (putative TcV) showed few apparent substitutions (Table 2), perhaps consistent with the recent origin of this DTU [66]. The sequence fragment we studied was outside the zinc binding domain of this metalloprotease, indicating selective forces can act on this protein independent of its core proteolyic function, perhaps through repeated exposure to host immunity.

It is important not to overlook the potential importance of multiclonal infections for parasitic disease, both as markers of population level factors such as parasite transmission, but also at the host level, including immunity and disease progression. In this study we have developed an amplicon sequencing approach to probe parasite genetic diversity within and among clinical CD cases to unprecedented depth. While our approach shows the power of this amplicon-seq to resolve diversity in clinical and congenital CD cases, it also highlights the potential biases that might be introduced with the addition of a PCR step. A tool that allows the accurate evaluation MOI would be valuable for tracking transmission rates at restricted disease foci (i.e. villages, outbreaks) in the context of measuring the success of intervention strategies. A similar tool could provide a powerful means of longitudinal tracking of *T*. *cruzi* infections in terms of disease progression, treatment failure and immunosuppression. Here we demonstrate that amplicon sequencing could have a role to play in this context. However, as sequencing costs decline and reference genome assemblies improve, whole genome deep sequencing, perhaps even of individual parasite cells, becomes and increasingly viable option as it already has for *Plasmodium sp*. [7,67].

## Supporting Information

S1 Fig TcGP63Ia and Ib amino acid alignments showing amplicon seq primer binding sites in relation to putative functional domains. Amino acid sequences are derived for those define by Cuevas and colleagues [24]. The colour key on the left hand side indicates primer binding sites and functional domains. The green shaded regions indicate the area covered by the Illumina paired end reads along each amplicon. The purple shaded central region indicates the area not covered.
(TIFF)

S2 Fig Bar plot of amplicon sequence data generated from control DTU mixes. Expected ratios of ND5 sequence types (far right) are compared to those recovered via amplicon sequencing. All three sequence types (I, II, III-VI) were recovered from all but the two most concentrated control mixes. However, the relative proportions of each sequence type derived from amplicon sequence data were radically different to that expected.
(TIFF)

S3 Fig Maximum likelihood phylogeny of 97% TcGP63I STs derived in this study and available *T*. *cruzi* and *T*. *cruzi marinkellei* TcGP63 paralogues. Homologous sequences were recovered from www.TriTrypDB.org via BLAST. The appropriate substitution model was defined as the transversion model with invariable sites plus gamma in Topali [68]. Abundant ST labels correspond with those indicated in Table 2. Branches are coloured by source DTU or red, for sequences generated in this study. Reference sequences TcGP3Ia and TcGP63Ib from the literature are also shown along side 97% sequence types generated in this study [24].
(TIFF)

S1 Appendix Quality filtered and assembled amplicon sequence data in FASTA format. (GZ)
