# Supplementary figures and images for: Deep Sequencing of the Trypanosoma cruzi GP63 Surface Proteases Reveals Diversity and Diversifying Selection among Chronic and Congenital Chagas Disease Patients
Source: PLoS Negl Trop Dis. 2015 Apr 7;9(4):e0003458. doi: 10.1371/journal.pntd.0003458 (PMC4388557; doi:10.1371/journal.pntd.0003458)

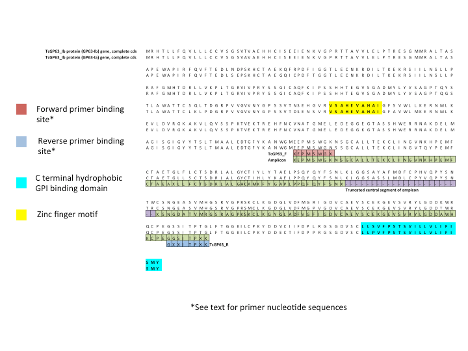

Supplement: S1 Fig — Amino acid sequences are derived for those define by Cuevas and colleagues [24]. The colour key on the left hand side indicates primer binding sites and functional domains. The green shaded regions indicate the area covered by the Illumina paired end reads along each amplicon. The purple shaded central region indicates the area not covered. (TIFF) [file pntd.0003458.s001.tiff]

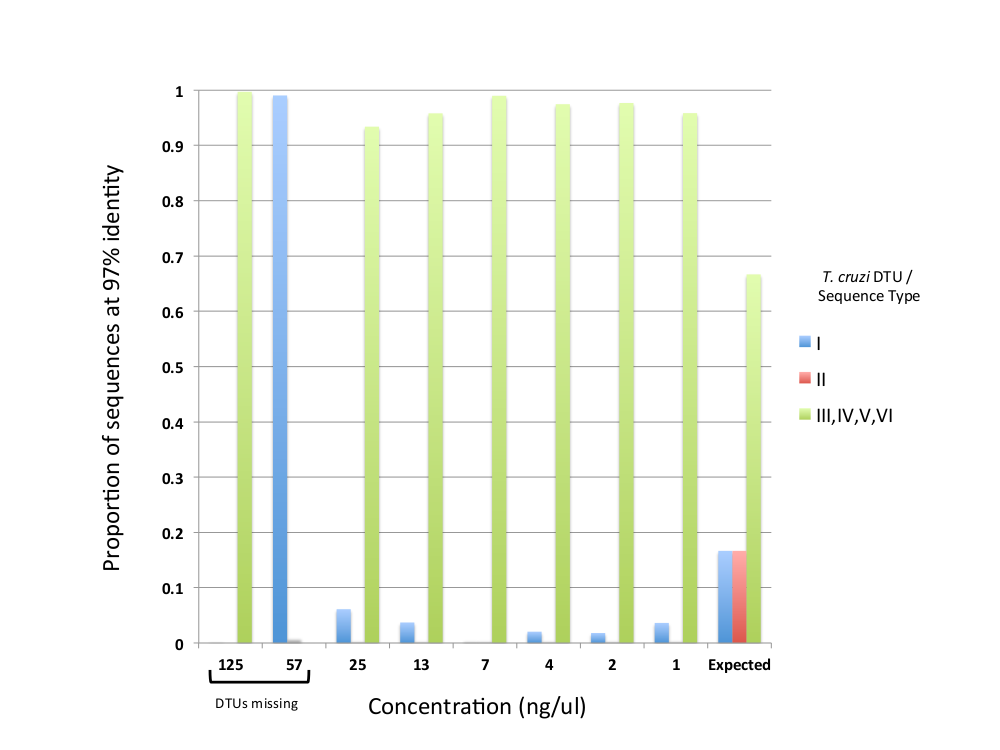

Supplement: S2 Fig — Expected ratios of ND5 sequence types (far right) are compared to those recovered via amplicon sequencing. All three sequence types (I, II, III-VI) were recovered from all but the two most concentrated control mixes. However, the relative proportions of each sequence type derived from amplicon sequence data were radically different to that expected. (TIFF) [file pntd.0003458.s002.tiff]

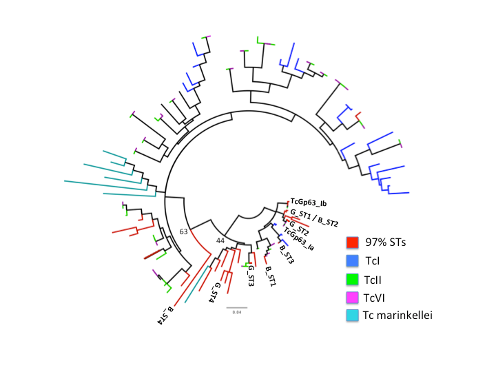

Supplement: S3 Fig — Homologous sequences were recovered from www.TriTrypDB.org via BLAST. The appropriate substitution model was defined as the transversion model with invariable sites plus gamma in Topali [68]. Abundant ST labels correspond with those indicated in Table 2. Branches are coloured by source DTU or red, for sequences generated in this study. Reference sequences TcGP3Ia and TcGP63Ib from the literature are also shown along side 97% sequence types generated in this study [24]. (TIFF) [file pntd.0003458.s003.tiff]
